# Turk Talk: human-machine hybrid virtual scenarios for professional education

**DOI:** 10.15694/mep.2018.0000266.1

**Published:** 2018-11-21

**Authors:** Michelle Cullen, Nishan Sharma, David Topps

**Affiliations:** 1 University of Calgary

**Keywords:** virtual scenarios, text-based chat, therapeutic communication, nursing education

## Abstract

This article was migrated. The article was marked as recommended.

Virtual scenarios provide a means for creating rich and complex online cases for health professional students to explore. However, the response options available to the learner are usually predefined, which limits the utility of virtual patients. Using artificial intelligence or natural language processing to accommodate such flexibility is expensive and hard to design. This project description lays out an alternative approach to making virtual scenarios more adaptable and interactive.

Using OpenLabyrinth, an open-source educational research platform, we modified the interface and functionality to provide a human-computer hybrid interface, where a human facilitator can interact with learners from within the online case scenario. Using a design-based research approach, we have iteratively improved our cases, workflows and scripts and interface designs. The next step is testing this new functionality in a variety of situations. This report describes the pilot implementation of this pilot project. It includes the background, rationale, objectives, learning and educational designs, and implications for software development.

The costs and time required to modify the software were much lower than anticipated. Facilitators managed text input from multiple concurrent learners. Learners noted a delay while waiting for the facilitator’s response, but denied becoming frustrated. The implementation and use of this new technique seems promising for training and assessment purposes related to developing effective communication skills. This report also explores the provisional implications arising from the study so far.

## Introduction

Virtual patients (VPs) are computer-based simulators of patient encounters for the purposes of instruction, practice, and assessment in health professional education. ( Ellaway et al., 2006) Virtual scenarios are a more generalized superset of problems and cases, which includes virtual patients, and which may not be clinically focused. For more on the differences, see https://openlabyrinth.ca/virtual-scenarios-overview. The strength of virtual scenarios comes from their qualities as practice simulators and from being accessible through almost any kind of computing device. ( Huwendiek et al., 2009) Their principal limitation however is that, without using complex and expensive artificial intelligence (AI), query responses need to be defined in advance. As a result, virtual scenarios are relatively limited in their ability to provide flexible representations of patient encounters or to incorporate adaptive instructor feedback. ( Huwendiek et al., 2009) Since all possible responses are pre-defined, there can be substantial cueing effects which may also be disadvantageous. ( McCarthy, 1966; Tabbers, Martens & van Merrienboer, 2004)

Although there has been some exploration of the use of text and speech recognition in virtual patients, it has proven to be complex, time consuming, and limited in its adaptability and range ( Danforth et al., 2009; Rizzo, Kenny & Parsons, 2011). Alternative approaches to online case simulators, such as standardized patients and mannequin simulators can be more effective in this regard but are resource intensive, lacking in scalability, and are of limited use in a distributed or networked educational environment. Thus, although the use of virtual patients has been associated with critical learning outcomes such as clinical judgment and decision-making ( Cook & Triola, 2009), their utility has been limited by the technologies that underpin them. In particular, we note the poor return on investment associated with using certain kinds of advanced technologies, such as AI ( Nirenburg et al., 2009), to provide natural language processing in a scalable and easy-to-use manner.

In this pilot project, we decided to explore the possibilities of human-computer hybrids as the basis for virtual patient interactions. Humans are better than computers at interpreting and responding within loosely defined or ambiguous contexts. For example, humans can better differentiate, based on context, when the language involves idiom, humour, colloquialisms, and local variations of medical jargon and abbreviations. However, one-to-one interactions, usually face-to-face such as those typical in Objective Structured Clinical Examinations (OSCEs) or standardized patients (SP) role plays, present problems of scalability and access, particularly for distributed educational programs. We need to make use of web-based media to provide access and scalability, while engaging human agents to manage episodes of semantically rich communication between learner and activity. The challenge then is how to leverage human pattern recognition and language processing abilities across multiple concurrent users in a distributed, networked environment.

There are examples of this type of resource leveraging in the commercial world with customer relationship management systems that use remote operators, chat-based interaction, and decision support trees. A single operator can concurrently manage multiple clients, sometimes up to 12 at a time, depending on the tolerable delay in response and the cognitive load for the operator. ( Steindler, 2014; LiveChat Support, 2015) This reflects the story of the Mechanical Turk where a human performed tasks in the guise of a mechanical computer. ( Levitt, 2000) Amazon made reference to this in the naming of its Mechanical Turk service [www.mturk.com], something they describe as “artificial” artificial intelligence. In Amazon’s service humans undertake semantically rich tasks that involve judgement and wisdom, which machines cannot do, to respond to requests mediated through an online system. Building on this principle, we developed and tested ways of using human agents to respond to learners within virtual scenario activities. This paper describes our progress to date on the development, testing, and utility of human-driven natural language functionality in the design of virtual scenario cases, using the open-source OpenLabyrinth platform ( Ellaway, 2010).

The aim of the study was twofold:

1. To develop Turk Talk functionality within OpenLabyrinth, so learners undertaking a virtual scenario activity can engage, by typing free-form answers, questions, hypotheses, or suggestions with one or more facilitators in a conversation.
2. To test these new tools and techniques (for functional quality, performance, and utility) across a wide range of settings, and to incorporate the findings into the software quality assurance and improvement process.

This paper describes the lessons learned during the initial program design and software development phase. It also describes how we have used these learning designs so far in the hope that others will be encouraged to try this approach.

## Methods

Because there are so many intersecting variables and unknowns to such a development, we needed an iterative cycle of design and testing that looks at how teachers and learners are guided by and interact with this software adaptation. We have therefore adopted a design-based research approach. ( Design-Based Research Collective, 2003) See Appendix 1 for more information about this approach.

*Setting:* The program is being conducted at the University of Calgary with testers, students, and faculty members from the Faculty of Nursing and the Cumming School of Medicine. We have also been collaborating with fellows of the Royal College of Surgeons in Ireland, who are interested in exploring the educational opportunities afforded by this approach.

*Participants:* Mental health virtual patient scenarios were designed for fourth year nursing students, enrolled in mental health and addictions elective course, in the Bachelor of Nursing (BN) program, in the Faculty of Nursing at the University of Calgary. Participation in the virtual patient scenarios and program evaluation will be required as part of the course curriculum. Facilitators volunteered their time and included faculty members and clinicians who had experience in caring for individuals and families experiencing mental illness and addictions and were aware of the mental health and addiction learning outcomes for the BN program. We also enlisted medical education research faculty from the Cumming School of Medicine to assist with the learning designs and program evaluation components. These same faculty members were asked to participate in initial face validity testing of the case materials.

*Intervention:* Learners are presented with complex branching clinical pathways, enquiry and feedback mechanisms, linked discussion forums, and externally linked educational resources. The OpenLabyrinth platform can support multiple concurrent users in a networked environment. Virtual patient cases can be made available to individual or groups of learners by assigning passwords using the Scenario Manager. Rapid reporting and simple messaging systems are already inherent in the software design of OpenLabyrinth.

The learning design and concept mapping tools used to construct a set of pathways and responses were also used to generate a standardized set of scripts and decision support trees for the facilitators. For a sample script, see our example case. ( Topps, Cullen & Sharma, 2015)

**Figure 1.**
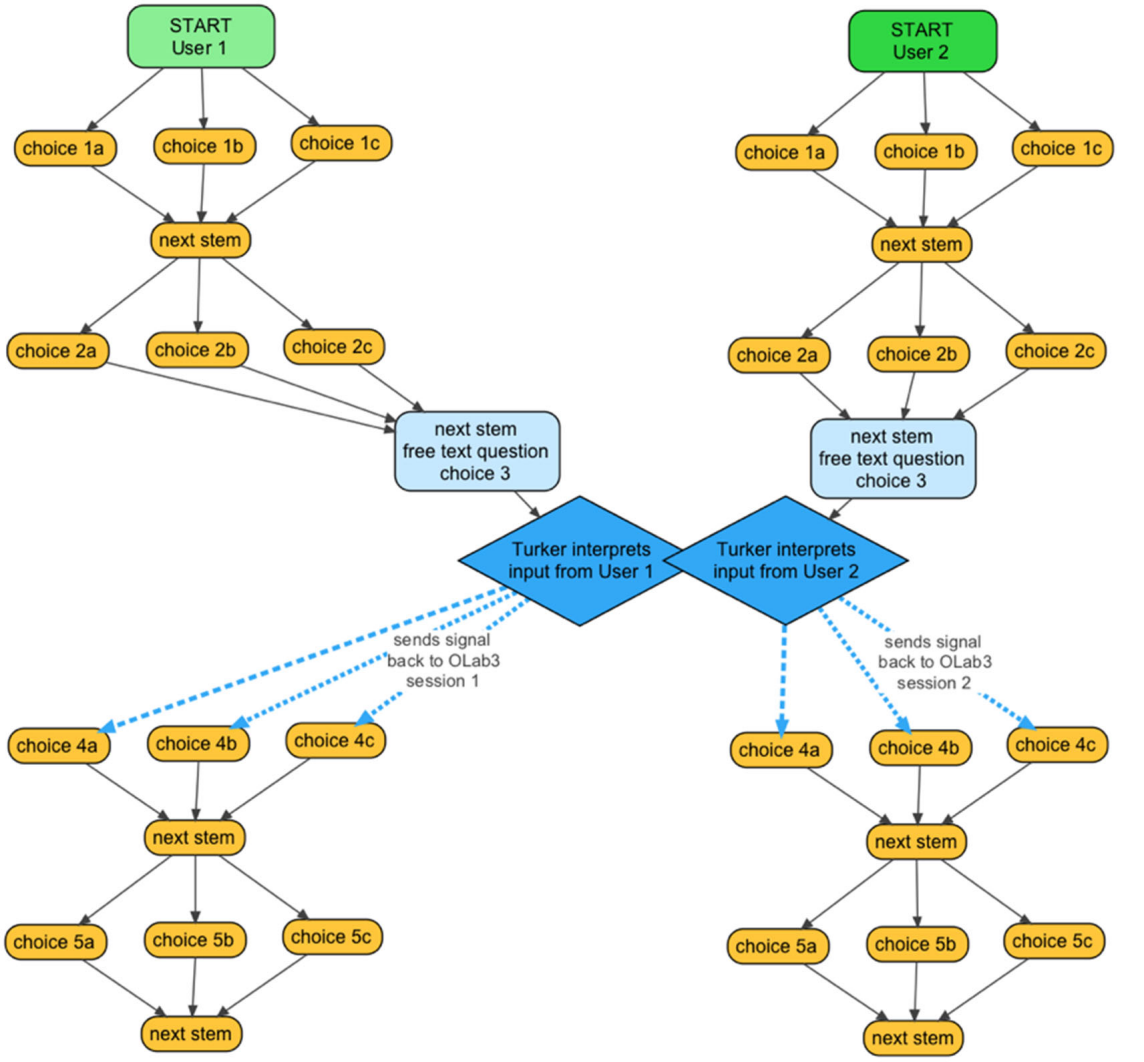
workflow or information flow in a Turk Talk case

While playing the scenario, learners independently navigate their way along a narrative pathway, interacting with the facilitator at interspersed intervals through the problem or case. Figure 1 illustrates two pathways, running in parallel through a set of nodes. The facilitator or “Turker” interacts in real-time with the learners at Turk Talk nodes (marked blue above), and moves learners to the next node in their narrative paths. The two learner pathways are concurrent and independent of each other, they each interact with the same Turker, who oversees the progress and responses for each learner.

OpenLabyrinth tracks all actions, mouse clicks, and chat responses, for each user with timestamps. These metrics are used to track performance and decision-making amongst learners. The metrics can also be used to track performance of the Turkers, or of the decision trees and scripts that they utilize. Examination of these analytics affords case designers with an internal quality improvement and audit mechanism. These metrics lend themselves both to quantitative analysis of numeric or categorical inputs, and qualitative analysis of the text responses. OpenLabyrinth provides some analytics tools through its own reporting functions, but data can also be exported to Excel or queried directly using standard SQL database tools.

OpenLabyrinth was extended to provide the additional functions required with this approach. More specifically, new mechanisms were developed to support:

1. concurrent display of multiple learner session pathways to Turkers
2. rapid collating of new user-generated input from selected Turk Talk input nodes
3. ability for Turkers to respond to each learner by text chat message
4. ability for Turkers to direct each learner on to next appropriate step in pathway

**Figure 2.**
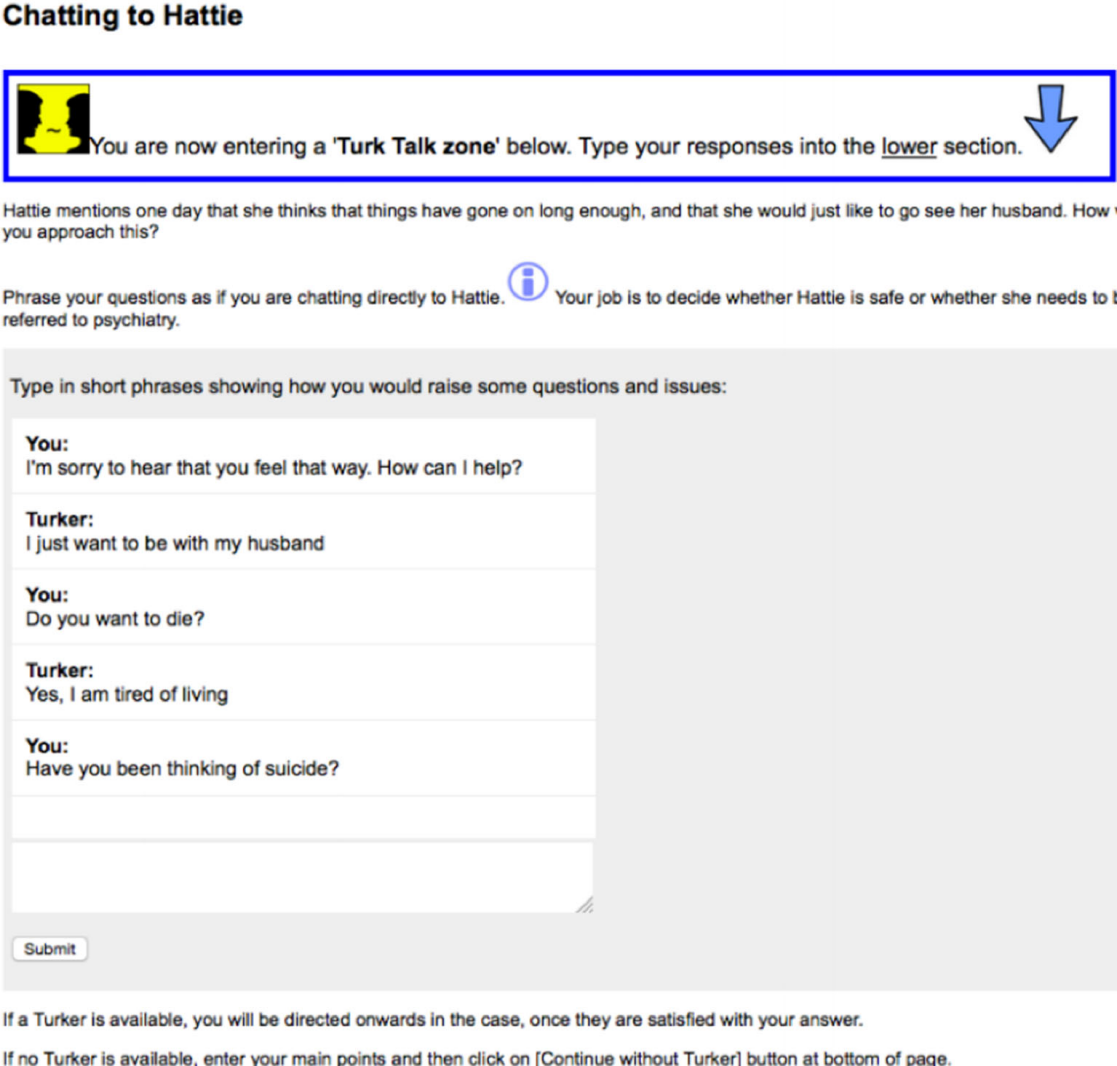
Turk Talk chat panel

Figure 2 below shows what the learner sees when they encounter a Turk Talk chat panel. The learner enters responses in the bottom section of the chat panel, and the conversation thread of the learner and Turker scrolls upwards in the top section of the panel. Previous entries can be viewed by scrolling back. Common responses can be scripted and inserted by the Turker using macros, which helps to keep up with multiple concurrent conversations.

**Figure 3.**
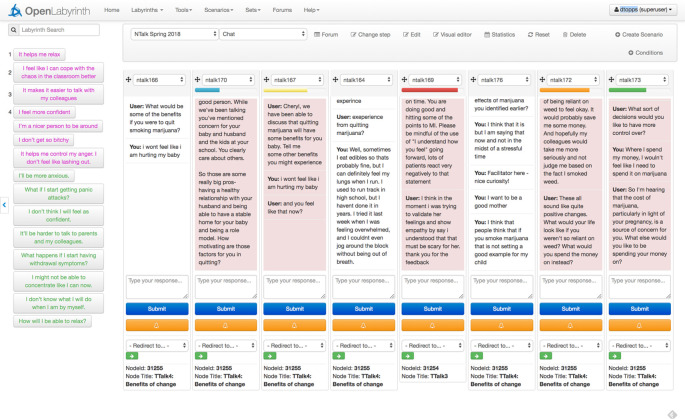
Turker’s overview of 8 chat streams

Figure 3 below shows the screen used by the Turker. There are up to eight columns, each with a conversation thread. Pink columns are awaiting input from the Turker. The colored timer bars above each pink column represent how long each user has been waiting, and in which order they should be attended: red has been waiting longest then orange, yellow, green and blue. Colors are reassigned as learners are dealt with in turn. Below the chat columns are optional target nodes that the Turker can redirect the learner onwards to, once they are satisfied that this Turk Talk interactive chat section has been completed.

### Extending OpenLabyrinth

**Figure 4.**
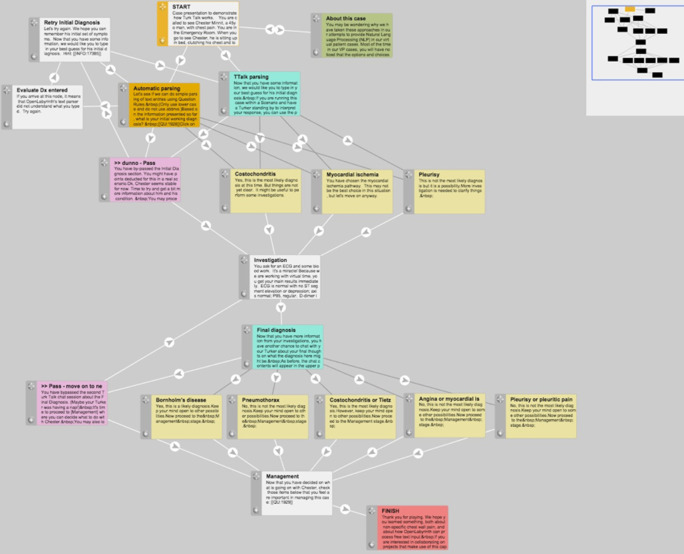
concept map from OpenLabyrinth visual editor, showing logic pathways

The pilot patient case designs in OpenLabyrinth employed a linear case design, with branching pathways. OpenLabyrinth provides multiple ways of presenting choices to users within an individual page or Node, including multiple-choice questions, drag and drop lists, etc. However, one particular case design that is popular amongst our case authors is using dandelions (multiple options or petals returning to the same central point) and branched sets of pathway choices. This allows the learner to choose between complex related concepts, receiving detailed feedback as they proceed. For an illustration of how this works, see Figure 4 below for an example concept map.

In this concept map, extracted from our first Turk Talk case design, the flow is from top downwards. The aquamarine nodes are Turk Talk nodes where the learner interacts with the Turker. The light-yellow nodes are the possible destination points to which the Turker can send the learner. The dark grey links signify hidden paths that are not immediately apparent to the learner as a choice but that the Turker can direct them along. Using dandelions and branched sets in this pathway design has the advantage that OpenLabyrinth can track user activity in much greater detail, examining such parameters as time taken to respond, node order precedence, complexity of the navigational pathway chosen by the learner, and efficiency of case navigation. These features provided real-time feedback to Turkers, such as where the learner was in the case and what choices were available at any given decision point. For those who are interested in how the user interface and learning design works, we refer them to our online tutorial, embedded in the first of our Turk Talk cases. ( Topps, Cullen & Ellaway, 2016)

We designed two separate virtual patient cases for our initial two trial runs with interested Turkers and faculty members:

1. Our first case design was short and straightforward so that we could run several iterations of the case and allow faculty members an opportunity to act in the Turker role. We incorporated some simple rule-based text processing into the case as a comparator so that Turkers and testers could get a feel for the expected machine-generated and human-generated responses. We recorded these sessions, took field notes, and debriefed after each simulation. We sought group input about changes for the software interface and the virtual patient case designs.
2. In our second trial, we made the virtual patient case design more complex with the intention of “stress testing” the Turker. The interface was set up to accommodate up to eight concurrent learners. We set up our session so that all eight learner slots were taken. Initially all eight learners were managed by one Turker, and then the learners were divided between two Turkers. We intentionally placed the most complex interaction nodes towards the end of the case so that the learners would arrive at this point in a staggered manner. This was done to decrease the likely lag time while learners waited for the Turker to respond, and to determine if it would be confusing for the Turker to have learners interacting with them at many different stages within the case.

We adopted an agile design process, rapidly incorporating feedback and suggestions from the testers into the software and case designs. We analyzed the case activity metrics, using the built-in timestamps and pathway metrics available in OpenLabyrinth, and by directly querying the underlying SQL tables, as noted above. This allowed us to further refine our case design performance in an iterative manner.

The Conjoint Health Research Ethics Board (CHREB), University of Calgary regards this phase as Program Evaluation. For the next phase, with direct investigation of learner performance, CHREB has granted ethical approval to carry out this study. (Certificate REB17-1950.)

## Results

So far,we have built the basic functionality in OpenLabyrinth, and we have formally tested this in two trial runs with faculty members from Medicine and Nursing. The software programming costs of implementing this design were 74% less than projected, and the development schedule was complete two months before anticipated delivery.

Participants in the trial sessions commented favourably on how easy the interface was to use. Small refinements to enhance efficiency and responsiveness have been introduced but the overall design has proven sound. Our test “learners” (faculty members acting in learner mode) have commented that they were impressed with how quickly they received a Turker response. Average response time was 31.1 (+/- 22.1) seconds. They were expecting delays of 1-2 minutes, based on previous experiences with similar chat-style interfaces in commercial technical support settings. Initially we were concerned that learners would become bored while waiting for a response from the Turker, so we designed several additional learning opportunities into our virtual patient cases for the second trial run. For example, while learners were waiting for a response, they could leave a comment about the case, or even play tiny games related to the content of the case. Learner feedback indicated the length of time spent waiting for a response did not warrant the need for additional activities. Our next steps will be to employ this learning design in real courses with real learners.

The scalability of this approach will be crucial to many centres. Early indications are that this approach is feasible: a skilled facilitator may be able to handle up to eight concurrent users. As we continue with this approach in the next phases of the study with learners from the Faculty of Nursing, more data on this will be available in future reports.

Our labyrinth authors learned to be sparing in their use of this Turk Talk interface. While it was tempting to make a large number of nodes into free-text or Turk Talk nodes, this starts to resemble the text-based navigation of Interactive Fiction. ( Derksen & Zundark, 2011) Most users find such a navigation interface to be slow and frustrating. By focusing on the approximately 10% of choices where it is important that the learner is not cued to the possible or expected responses, the navigation flow can be kept efficient and provide challenging and contextual learning. This usually translates as one or two Turk Talk interactions per case.

By restricting the use of Turk Talk nodes to a few key locations within the narrative pathway, our authors also found that this accelerated the case design process, both in the initial design and also the debugging phases of case design. The time spent on case authoring averaged 94 minutes (+/- 32 mins) over the six-case series.

Our initial trial runs also highlighted the importance of properly preparing the Turkers. We provided them with concept maps illustrating the allowed pathways within the map, along with scripts and tables of acceptable and unacceptable learner responses. ( Topps, Cullen & Sharma, 2015) See http://demo.openlabyrinth.ca/renderLabyrinth/index/606 for a live example of a Turk Talk case and associated files. We also worked with the Turkers to create shortcut text macros for frequently used responses such “tell me more” or “you need to read the question again”. The current format of these Turker scripts is cumbersome and we anticipate further refinements to this process as we iteratively improve these in real learner sessions.

Although the original design had intended a one-to-eight ratio between the Turker and their assigned learners, during our second trial run, we were pleased to discover that it was possible to run two Turkers concurrently on separate terminals, each monitoring the same group of learners. Each Turker could see what the other was doing, the progress for all learners, and learners could receive responses from either Turker. This process went smoothly, with no apparent lag in the software. Functionally, workflow was more effective if the Turkers were assigned and individually responsible for particular learners, rather than their responses overlapping. In the user interface, visual grouping of learners according to each Turker’s preferences were created. In addition, a visual progression of multiple Turkers and their assigned groups was created and allowed for more complex learning designs. For example, a more experienced teacher can supervise novice or front-line Turkers. As part of our future testing, we will further explore how scalable this approach is and what complexities arise as additional layering into the supervisory structures is introduced. Additional functionality where learners can collaborate through sharing their responses may also be explored in future versions.

## Discussion

In comparison with traditional linear virtual patient case designs, our testers appreciated the greater flexibility of design afforded by branching case designs and dandelions. As with other virtual patient studies that have used OpenLabyrinth, branched case designs with pathway options that reflect realistic decision points and clinical choices are much preferred over simple linear case designs, which are often disparagingly paraphrased as “page turners”. ( Woodham et al, 2015; Topps et al, 2014)

The provision of an interface that resembles natural language processing appears to make the case design process much easier, avoiding the need for complex logic designs or choice restrictions. This has been attempted before with Interactive Fiction software ( Derksen & Zundark, 2011) and with very sophisticated designs such as the Maryland Project ( Nirenberg et al, 2009) but our approach seems to be simpler, more affordable and more efficient.

We anticipate being able to use this approach in a variety of learning designs in upcoming studies:

Learning Designs

1. Two to eight concurrent learners working on the same OpenLabyrinth scenario or set of linked VP cases, with key sections in the case map designated for real-time chat sessions. Turkers can identify how learners are moving through the scenario and when they begin and complete a Turk Talk session by the progress bar at the bottom of each learner’s Turk Talk column. The Turker reads and interprets the learner’s text and responds. A Turker may respond with a combination of possible responses provided by the OpenLabyrinth decision support pathways, or with a free text response. Pre-scripted macros are similar to pre-determined responses to SMS text messages, and are used for common responses. These macros decrease the response time by omitting the time it takes to type out a response in full. Free text responses can capture the unique nuances of language and enable the Turker to respond in a contextually relevant manner. The Turker then directs learners to the next appropriate Node. The learner continues with the case map from that Node in the usual manner of virtual patient case navigation, until the case ends or the next round of Turk Talk interpretation is required.
2. Bookending over time, with early simple cases, progressing to more complex and intense interactions, and possibly ending in a more sophisticated full Standardized Patient experience, allows teachers to scaffold diverse concepts and provide integrated learning experiences in a safe environment where real patients are not being harmed and students have opportunities to debrief and reflect on their strengths and areas for improvement.
3. When parallel participants are playing the cases asynchronously, there may be some delay in how quickly the Turkers are able to respond to learners. Learners were informed that there may be delays of varying lengths. From the feedback, we noted that the delay was less bothersome when learners knew about the possibility before beginning the scenario. We also know that with simple semi-synchronous word games played on mobile phones, like “Words with Friends”, that delays of minutes or even hours are tolerated, depending on the context and expectations of the players.
4. Learning to communicate with minority ethnic or cultural groups, or with those experiencing diverse health concerns, such as mental illness, requires different communication conventions (i.e. less or no eye contact, different phrasing or idioms or concepts of time). It can be challenging to create learning experiences that simulate these types of interactions, especially with a shortage of skilled facilitators or SPs. We intend to explore how virtual patient scenarios might address these learning needs; however, we also intend to explore how the Proteus Effect, first demonstrated in the work from Stanford ( Yee & Bailenson, 2007), may influence learners. The Proteus Effect occurs when the learner begins to act in accordance with the presentation of their avatar in various on-line games and simulated activities ( Yee & Bailenson, 2007). For example, converting a large, ebullient, domineering alpha-male preceptor into a shy retiring female patient by use of avatars, plus pre-recorded response phrases, was shown to create interesting changes in participant behaviour in their studies. Our design affords a simpler, cheaper approach to explore such educational facets in cultural sensitivity training.
5. Scaling up to larger numbers of concurrent learners will be explored, using a Tier 1 and Tier 2 support model, typically seen in the Customer Relationship Management systems in a tech-support office. Tier 1 responses are more simplistic and can largely be patterned into common phrases after two to three iterations of the Scenario. This allows for the possibility of having non-expert or non-clinical facilitators. More complicated questions can then be escalated to Tier 2 clinical supervisors who can provide more contextually relevant responses. Geographic location is irrelevant, allowing home-based workers or outsourced labour to be employed.

We are interested in hearing from others who may wish to explore this approach in their virtual patient scenario designs. The source code will be incorporated into upcoming versions of OpenLabyrinth, which is free and open-source on GitHub ( Topps et al., 2015). But for those teams who wish to try this out in a collaborative fashion, we may be able to provide guidance on case and scenario writing challenges. Please contact us via https://openlabyrinth.ca/contact/ and we can set you up with a demo login.

## Take Home Messages

• Turk Talk, a text-based chat, introduced as part of virtual scenarios, provides a practical, scalable, affordable means of providing natural language interpretation into scenario navigation.
• Learners and teachers readily adapt to this new interaction mechanism for virtual scenarios.

## Notes On Contributors

• Michelle Cullen, BN, is currently defending her Masters in Nursing thesis at the Graduate Program in Nursing at the University of Calgary.
• Nishan Sharma, MSc EdD, is Education Lead at W21C, and a Research Assistant Professor in the Department of Community Health Sciences at the University of Calgary. After over a decade’s experience teaching undergraduate science, nursing, and medical students, he earned his doctorate in education in 2009, where his work focused on curricular change in medical schools. His current research interests focus on teaching and learning in medicine and the integration of information technology into medical education.
• David Topps, MB ChB, is Medical Director in the Office of Health & Medical Education Scholarship (OHMES) at the University of Calgary. He has over two decades of experience innovating in technology based learning, virtual scenarios, assessment techniques and mobile learning. orcid.org/0000-0003-3593-544X

## Appendices

### Appendix 1: Perspectives on Design Based Research

Design-based research (DBR) is a practical research methodology that can bridge the chasm between research and practice in formal education and is defined as ‘being situated in a real educational context’. ( Anderson & Shattuck, 2012) The approach focuses on the design and testing of an educational interaction.

Many readers, upon encountering an article that reports on findings from a Design Based Research (DBR) approach, are thrown off by the departure from the traditional positivist scientific method. “Where is the control group? What was the actual intervention? What is your dependent variable?” are commonly raised questions.

Educational research is often conducted in a complex environment, with many confounding factors, interacting in complex systems. DBR offers an approach which accommodates some of this complexity, gathering data through mixed quantitative and qualitative methods, and looking at the multiple variables and outcomes that are emergent from these multifaceted systems.

Intrinsic to DBR is that it is an iterative process, not a single intervention. Based on successive rounds of findings, the researchers refine their processes, tools and theories. Researchers and participants are engaged interactively in the study design. Contributions from study subjects may influence subsequent iterations.

Some have raised concerns that this creates local solutions that are not generalizable. Others have countered that isolated, idealized laboratory experiments with highly controlled conditions also bear little relation to the practicalities of real-world implementation. Rather than proselytizing over principles, it is more constructive to value each approach for its strengths and what it brings to advancement of knowledge and practice.
