# Supplementary material for: Turk Talk: human-machine hybrid virtual scenarios for professional education
Source: MedEdPublish (2016). 2018 Nov 21;7:266. [Version 1] doi: 10.15694/mep.2018.0000266.1 (PMC10711943; doi:10.15694/mep.2018.0000266.1)
Supplement: Peer review history

# Reviewer report: Reviewer response for version 1

**DOI:** 10.21956/mep.19720.r29525

**Published:** 2018-12-06

**Authors:** Sateesh Babu Arja

**Affiliations:** 1 Avalon University School of Medicine

This review has been migrated. The reviewer awarded 4 stars out of 5 I thoroughly enjoyed reading this paper as I am the Director of Clinical Skills course at our university. A very well written paper on such a complex topic and easily understandable. We are using standardized patients and mannequin simulators at our university to teach Clinical Skills. It is exciting to see how can we use human-machine hybrid virtual scenarios in medical and health professions. I commend authors for taking up such project. The literature review is relevant and updated. This is very useful for faculty members who are involved in teaching clinical skills and assessing health professional students.

Reviewer Expertise:

NA

No decision status is available

# Reviewer report: Reviewer response for version 1

**DOI:** 10.21956/mep.19720.r29524

**Published:** 2018-11-22

**Authors:** Ken Masters

**Affiliations:** 1 Sultan Qaboos University

This review has been migrated. The reviewer awarded 4 stars out of 5 A very well written paper on a relatively complex topic. The problems are clearly explained and the solutions well-presented. The authors have done extremely well in navigating the explanations, ensuring that that they do not over-simplify the technical aspects, and yet ensuring that the general reader is not lost in computer jargon. The authors have also used the Discussion area to give an extremely detailed reflection on the trial runs. Some readers may find it a little too detailed, but I commend the authors on their analysis. More than that, moreover, is the way in which the authors have approached the presentation of the paper:1. Using the Appendix for further theoretical discussion of the methodology – those who are knowledgeable of the approach can move through the paper more fluidly, and those who are unfamiliar have the opportunity to explore in some more detail.2. Supplying the ancillary data and details (links to external websites, inviting further participation by readers) takes the paper beyond the narrow scope of a paper in an academic journal, and uses the medium of the journal in ways in which it was intended. This paper is a valuable example of how 21st century publishing media should be used.Overall, a valuable contribution to medical education literature.Some issues:• In the abstract: “but denied becoming frustrated” this is rather awkward phrasing.• Personally, I would like to have seen a short conclusion drawing the strands together, particularly because of the complexity of the topic. Similarly, the Take-Home messages could also be fleshed out a little more, as they sell the paper short.• The authors should note a limitation of the system: the need for human tutors. Although this is integral to the success of the system, many students would approach a computer system with the idea of 24/7 access, either because they would be in a mind-set of interacting with computers only, or because they are familiar with commercial systems utilising international centres. Learners’ expectations of realistic “teaching hours” would have to be addressed beforehand and managed carefully.

Reviewer Expertise:

NA

No decision status is available
